# Prognostic significance of albuminuria in elderly of various ages with diabetes

**DOI:** 10.1038/s41598-023-32305-0

**Published:** 2023-05-01

**Authors:** Subin Hwang, Kyungho Lee, Jeeeun Park, Do Hee Kim, Junseok Jeon, Hye Ryoun Jang, Kyu Yeon Hur, Jae Hyeon Kim, Wooseong Huh, Yoon-Goo Kim, Jung Eun Lee

**Affiliations:** 1 Division of Nephrology, Department of Internal Medicine, Seoul Paik Hospital, Inje University College of Medicine, Seoul, South Korea; 2 Division of Nephrology, Department of Internal Medicine, Samsung Medical Center, Sungkyunkwan University School of Medicine, Seoul, South Korea; 3 Division of Endocrinology and Metabolism, Department of Internal Medicine, Samsung Medical Center, Sungkyunkwan University School of Medicine, Seoul, South Korea

**Keywords:** Diabetes, Kidney diseases, Renal replacement therapy, Diabetic nephropathy

## Abstract

It remains uncertain whether albuminuria can identify elderly patients with diabetes at a high risk of incident end-stage kidney disease (ESKD) or mortality. 3065 patients (aged ≥ 65 years) with type 2 diabetes were included. We examined the association between albuminuria stages (normoalbuminuria, A1; microalbuminuria, A2; and macroalbuminuria, A3) and the risk of incident ESKD and all-cause mortality for each age group (65–69, 70–74, and ≥ 75 years). A2 and A3 were observed in 25.5% and 9.4% of the subjects, respectively. For A1, A2, and A3, the probabilities of ESKD at 8 years were 1.0%, 6.3%, and 29.7% (*P* < 0.001 for all), and the all-cause mortality was 13.1%, 27.4%, and 31.7% (*P* < 0.001 for A1 vs A2, *P* < 0.001 for A1 vs A3), respectively. Albuminuria stages were independently associated with an increased risk of ESKD [fully adjusted hazard ratios (HR): 3.650 (1.987–6.702) for A2, 10.404 (5.706–18.972) for A3 vs. A1]. The HRs of all-cause mortality were 1.742 (1.411–2.153) for A2 and 1.810 (1.344–2.441) for A3. The associations between albuminuria stages and the risk of ESKD and all-cause mortality were consistent across all age groups. Even microalbuminuria is also a risk factor for incident ESKD and mortality in elderly patients with diabetes.

## Introduction

The number of people with diabetes is expected to increase substantially^1^. The prevalence of diabetes increases with age, reaching one-quarter of those aged 65 years or older^2^. Diabetes is accompanied by several complications that contribute to an increased risk of cardiovascular disease (CVD) and mortality, and these complications are prevalent in elderly patients^3^. Kidney function declines naturally with aging and is accelerated in people with diabetes^4,5^. Consequently, patients over 65 years of age with diabetes show a higher prevalence of decreased kidney function and diabetic kidney disease (DKD) than younger patients^6^.

In addition to a decreased estimated glomerular filtration rate (eGFR), albuminuria is a key component of DKD and a robust prognostic factor for kidney and cardiovascular outcomes in patients with diabetes^7–11^. Renin–angiotensin–aldosterone system (RAAS) blockers, the cornerstones of DKD treatment, delay disease progression via reduction of albuminuria. However, recent epidemiological studies have shown that albuminuria-associated risk of all-cause mortality and cardiovascular mortality is attenuated in elderly patients with diabetes^12,13^. Moreover, there are limited data regarding whether albuminuria stage is associated with an increased risk of end-stage kidney disease (ESKD) in elderly patients with diabetes.

Understanding the consequences of albuminuria in elderly patients with diabetes is the first step for future research efforts as well as for prioritizing and individualizing treatment for kidney protection. In this longitudinal study of elderly (≥ 65 years) patients with diabetes, we evaluated the effects of albuminuria on incident ESKD and all-cause mortality. We also assessed the effects of stratifying the patients according to age group.

## Results

Of the 3065 elderly patients with diabetes in this analysis, 1389 (45.3%) were aged 65–69 years, 976 (31.8%) were aged 70–74 years, and 700 (22.8%) were aged 75 years and older. The mean (SD) age was 71.1 (5.0) years, and 52.9% were male (Table 1). The mean duration of diabetes was 13.4 (8.7) years, and the mean glycosylated hemoglobin (HbA1c) level was 7.2 (1.1)% at baseline. The prevalence of albuminuria increased progressively in older age groups (in patients aged 65–69 years, 70–74 years, and ≥ 75 years, the prevalence was 22.1%, 24.9%, and 33.0%, respectively, for microalbuminuria and 8.4%, 9.8%, and 10.6%, respectively, for macroalbuminuria, *P* < 0.001 for trend). Decreased kidney function was much more prevalent with aging (in patients aged 65–69 years, 70–74 years, and 75 years and older, the prevalence was 13%, 19.9%, and 30.0%, respectively, for 45 ml/min/1.73 m^2^ ≤ eGFR < 60 ml/min/1.73 m^2^; 4.9%, 8.5%, and 14.4%, respectively, for 30 ml/min/1.73 m^2^ ≤ eGFR < 45 ml/min/1.73 m^2^; and 1.7%, 2.0%, and 3.7%, respectively, for 15 ml/min/1.73 m^2^ ≤ eGFR < 30 ml/min/1.73 m^2^, *P* < 0.001 for trend). The mean Charlson Comorbidity Index (CCI) score was 2.24 (1.24); 2.7% had heart failure (HF), 23.1% had CVD, 42.3% had end-organ damage, and 9.9% had a history of cancer. The prevalence of all comorbidities progressively increased with age. There was no difference in the frequency of angiotensin-converting-enzyme inhibitors (ACEIs) or angiotensin II receptor blockers (ARBs) use among the three age groups (*P* = 0.751). A total of 472 patients (15.4%) were treated with insulin, and 2,109 (68.8%) with metformin. HbA1c levels were similar among three the age groups (*P* = 0.529).

**Table 1:** Baseline characteristics in elderly (≥ 65 years) patients with diabetes according to age.

	Total	65–69 years	70–74 years	≥ 75 years	*P* value
	(n = 3065)	(n = 1389)	(n = 976)	(n = 700)	
Age, year	71.1 ± 5.0	66.9 ± 1.4	71.8 ± 1.4	78.6 ± 3.3	< 0.001
Male	1621 (52.9%)	784 (56.4%)	517 (53.0%)	320 (45.7%)	< 0.001
BMI, kg/m^2^	24.8 ± 3.1	24.8 ± 2.9	24.9 ± 3.2	24.6 ± 3.3	0.148
SBP, mmHg	127.0 (119.0–136.5)	126.0 (118.0–134.5)	127.0 (118.5–136.5)	130.5 (121.5–139.5)	< 0.001
Duration of diabetes, year	13.4 ± 8.7	12.4 ± 8.0	13.8 ± 8.9	14.8 ± 9.7	< 0.001
Urine ACR, mg/gCr	14.16 (6.00–54.46)	11.17 (5.26–43.51)	14.69 (6.16–55.94)	21.96 (8.16–81.26)	0.144
ACR < 30	1997 (65.2%)	965 (69.5%)	637 (65.3%)	395 (56.4%)	< 0.001
ACR 30 to 300	781 (25.5%)	307 (22.1%)	243 (24.9%)	231 (33.0%)	
ACR > 300	287 (9.4%)	117 (8.4%)	96 (9.8%)	74 (10.6%)	
Baseline eGFR, ml/min/1.73 m^2^	68 ± 17	72 ± 16	67 ± 16	60 ± 16	< 0.001
eGFR ≥ 60	2159 (70.4%)	1117 (80.4%)	679 (69.6%)	363 (51.9%)	< 0.001
45 ≤ eGFR < 60	584 (19.1%)	180 (13.0%)	194 (19.9%)	210 (30.0%)	
30 ≤ eGFR < 45	252 (8.2%)	68 (4.9%)	83 (8.5%)	101 (14.4%)	
15 ≤ eGFR < 30	70 (3%)	24 (1.7%)	20 (2.0%)	26 (3.7%)	
HbA1C, %	7.2 ± 1.1	7.2 ± 1.1	7.2 ± 1.1	7.2 ± 1.1	0.529
HDL-cholesterol, mg/dl	49 ± 13	49 ± 13	49 ± 13	49 ± 14	0.748
LDL-cholesterol, mg/dl	98 ± 29	98 ± 28	97 ± 28	98 ± 30	0.415
CCI score^a^	2.24 ± 1.24	2.13 ± 1.12	2.25 ± 1.21	2.43 ± 1.32	< 0.001
Comorbidity^a^					
Heart failure	84 (2.7%)	28 (2.0%)	27 (2.8%)	29 (4.1%)	0.019
Cardiovascular disease^b^	709 (23.1%)	255 (18.4%)	237 (24.3%)	217 (31.0%)	< 0.001
End organ damage^c^	1298 (42.3%)	525 (37.8%)	408 (41.8%)	365 (52.1%)	< 0.001
History of cancer	304 (9.9%)	138 (9.9%)	101 (10.3%)	65 (9.3%)	0.029
Medications					
ACEI or ARB	1998 (65.2%)	862 (62.1%)	665 (68.1%)	471 (67.3%)	0.004
Statin	1804 (58.9%)	809 (58.2%)	580 (59.4%)	415 (59.3%)	0.819
Insulin	472 (15.4%)	211 (15.2%)	156 (16.0%)	105 (15.0%)	0.824
Metformin	2109 (68.8%)	1010 (72.7%)	665 (68.1%)	434 (62.0%)	< 0.001
DPP-4 inhibitor	474 (15.5%)	241 (17.4%)	134 (13.7%)	99 (14.1%)	0.031
Sulfonylurea	1652 (53.9%)	726 (52.3%)	544 (55.7%)	382 (54.6%)	0.23
α-glucosidase inhibitor	942 (30.7%)	417 (30.0%)	295 (30.2%)	230 (32.9%)	0.381
Other medications for diabetes	456 (14.9%)	199 (14.3%)	147 (15.1%)	110 (15.7%)	0.689

Values are presented as number (%), mean ± standard deviation or median (interquartile range).

BMI, body mass index; SBP, systolic blood pressure; ACR, albumin to creatinine ratio; eGFR, estimated glomerular filtration rate; HbA1c, glycosylated hemoglobin; HDL-cholesterol, high-density lipoprotein cholesterol; LDL-cholesterol, low-density lipoprotein cholesterol; CCI, Charlson comorbidity index; ACEI, angiotensin converting enzyme inhibitor; ARB, angiotensin II receptor blocker; DPP-4 inhibitor, dipeptidyl peptidate-4 inhibitor; ICD-10, International Classification of Diseases 10th revision.

^a^CCI score and comorbidities using the ICD-10 codes.

^b^Myocardial infarction, cerebrovascular accident, transient ischemic attack, and peripheral vascular disease were combined into a cardiovascular disease based on whether any of the individual comorbidities were present.

^c^End organ damage was defined as the combination of diabetic neuropathy, diabetic retinopathy, and albuminuria.

**Figure 1:**
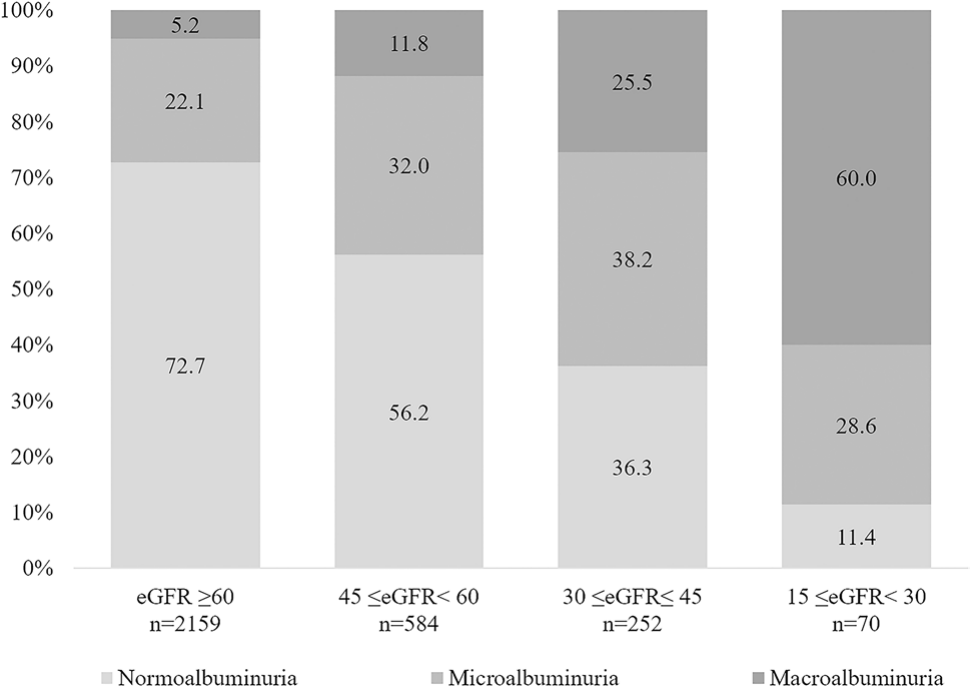
Prevalence of albuminuria stage according to estimated glomerular filtration rate (eGFR) in elderly (≥ 65 years) patients with diabetes. The prevalence of albuminuria varied markedly with eGFR and age. Overall, the prevalence of macroalbuminuria gradually increased, and the prevalence of normoalbuminuria decreased in the lower eGFR categories.

The prevalence of albuminuria varied markedly with eGFR and age (Fig. 1). Overall, the prevalence of albuminuria increased gradually as the eGFR categories worsened. Approximately 72.7% of all patients with an eGFR ≥ 60 ml/min/1.73 m^2^ had normoalbuminuria, and only 5.2% had macroalbuminuria. On the other hand, 11.4% of patients with a severely decreased eGFR (15 ml/min/1.73 m^2^ ≤ eGFR < 30 ml/min/1.73 m^2^) had normoalbuminuria and more than half had macroalbuminuria (60%).

**Figure 2:**
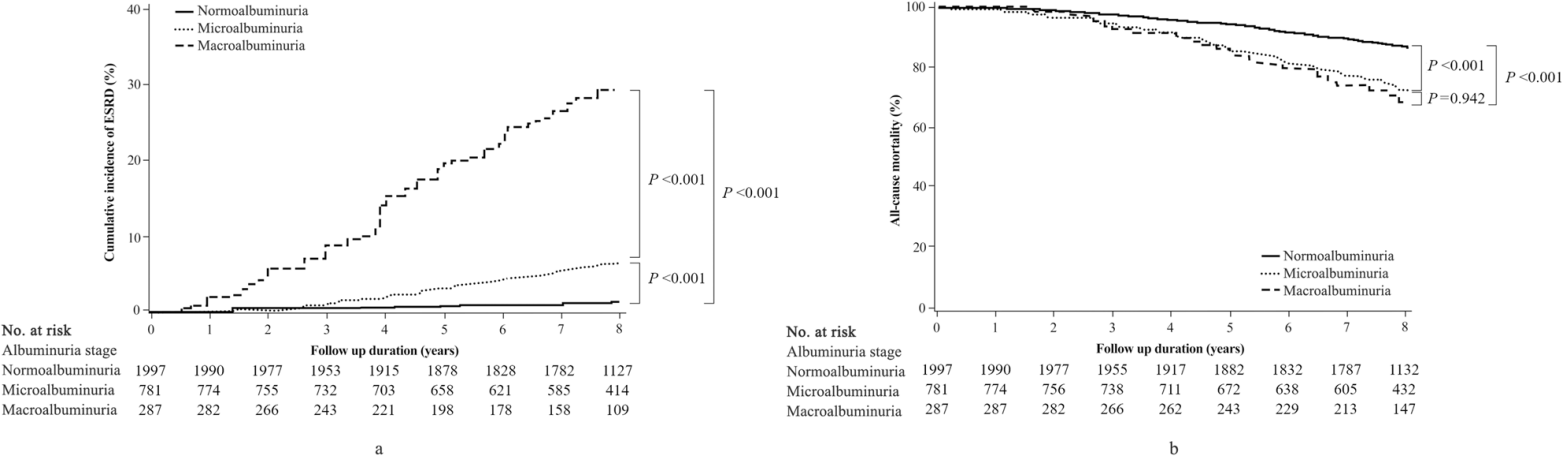
Cumulative incidence of end stage kidney disease (ESKD) according to albuminuria stage (with death as a competing risk) in elderly (≥ 65 years) patients with diabetes (**a**). The cumulative incidence of ESKD increased gradually with higher levels of albuminuria (*P* < 0.001 for all, A1 vs A2, A1 vs A3, and A2 vs A3). All-cause mortality according to albuminuria stage in elderly (≥ 65 years) patients with diabetes (**b**). Patients with normo albuminuria showed better survival rates than those with microalbuminuria or macroalbuminuria (*P* < 0.001 for A1 vs A2, *P* < 0.001 for A1 vs A3, and *P* = 0.942 for A2 vs A3). A1: normoalbuminuria (ACR < 30 mg/gCr), A2: microalbuminuria (ACR 30–300 mg/gCr), A3: macroalbuminuria (ACR > 300 mg/gCr). ACR, albumin-to-creatinine ratio.

The median follow-up time for outcomes was 92 (87–95) months. In 2017, 644 (21%) patients progressed to ESKD and 561 (18%) died. The cumulative incidence of ESKD increased gradually with higher levels of albuminuria (*P* < 0.001 for all; A1 vs. A2, A1 vs. A3, and A2 vs. A3) (Fig. 2a). The 5-year kidney survival rates were 91.3% for patients with A1, 86.0% for those with A2, and 84.7% for those with A3.

Regarding all-cause mortality, patients with normo albuminuria showed better survival rates than those with microalbuminuria or macroalbuminuria (*P* < 0.001 for A1 vs. A2, *P* < 0.001 for A1 vs. A3) (Fig. 2b).

**Figure 3:**
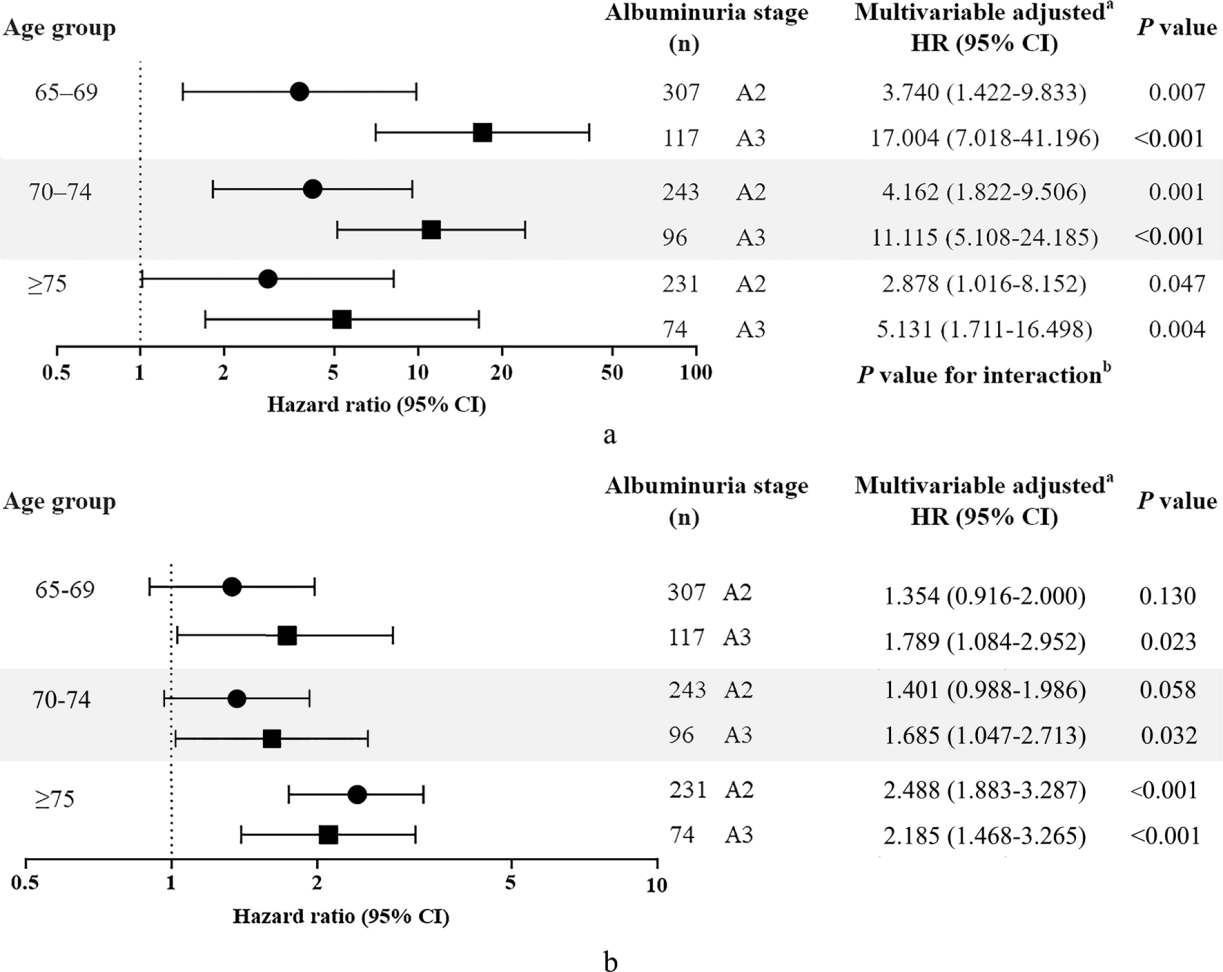
Hazard ratios of albuminuria stage for incident end stage kidney disease (ESKD) according to age (with death as a competing risk) in elderly (≥ 65 years) patients with diabetes (**a**). Albuminuria was a significant risk factor for incident ESKD in patients aged 65–69, 70–74, and ≥ 75 years. The higher the stage of albuminuria was the higher the risk of incident ESKD; this relationship was consistent across the different age groups. The hazards of albuminuria seemed to be attenuated in older groups. The interaction between albuminuria stages and age groups were not significant. Hazard ratios of albuminuria stage for all-cause mortality according to age (**b**). The increased risk of all-cause mortality with albuminuria was consistent in the three age groups. There were no significant interactions between albuminuria stages and age groups. ^a^Adjusted for baseline covariates, including age, sex, body mass index, systolic blood pressure, duration of diabetes, estimated glomerular filtration rate as a continuous variable, glycosylated hemoglobin, high-density lipoprotein cholesterol, low-density lipoprotein cholesterol, and comorbidities (CVD and heart failure). A1: normoalbuminuria (ACR < 30 mg/gCr), A2: microalbuminuria (ACR 30–300 mg/gCr), A3: macroalbuminuria (ACR > 300 mg/gCr). HR, hazard ratio; CI, confidence interval; ACR, albumin-to-creatinine ratio; CVD, cardiovascular disease.

To evaluate the effects of albuminuria on incident ESKD, we used Cox proportional hazards analysis, considering competing risks. Univariate analysis showed that albuminuria was associated with an increased risk of ESKD. After adjustment for age, duration of diabetes, eGFR, and other laboratory parameters, microalbuminuria was associated with a 3.650-fold increase in ESKD risk (95% confidence interval [CI] 1.987–6.702, *P* < 0.001). Macroalbuminuria was associated with 10.404 (5.706–18.972, *P* < 0.001) times the risk of ESKD (Table 2). Next, we compared the hazards of albuminuria for incident ESKD across different age groups (65–69, 70–74, and ≥ 75 years) (Fig. 3a). The hazards of albuminuria seemed to be attenuated in the older groups, but the associations between albuminuria categories and increased risk of ESKD were preserved in all the three age groups. Interaction analyses between the albuminuria categories and age groups were not significant.

**Table 2:** Risk factors of end stage kidney disease in elderly (≥ 65 years) patients with diabetes.

Variables	Univariate analysis		Multivariate analysis^c^	
	HR (95% CI)	*P* value	HR (95% CI)	*P* value
Age, year	1.027 (0.998–1.057)	0.068		
Female (vs. male)	0.987 (0.719–1.354)	0.936		
BMI, kg/m^2^	1.016 (0.959–1.076)	0.589		
SBP, mmHg	1.042(1.028–1.055)	< 0.001	1.017 (1.005–1.029)	0.005
Duration of diabetes (per 1 year)	1.049 (1.035–1.064)	< 0.001		
ACR < 30 mg/gCr	1		1	
ACR 30 to 300 mg/gCr	6.407 (3.811–10.772)	< 0.001	3.650 (1.987–6.702)	< 0.001
ACR > 300 mg/gCr	35.068 (21.535–57.105)	< 0.001	10.404 (5.706–18.972)	< 0.001
eGFR, ml/min/1.73 m^2^	0.923 (0.913–0.934)	< 0.001	0.944 (0.933–0.955)	< 0.001
HbA1c, %	1.386 (1.233–1.557)	< 0.001	1.188 (1.039–1.359)	0.012
HDL cholesterol, mg/dl	0.976 (0.962–0.990)	0.001		
LDL cholesterol, mg/dl	1.004 (0.998–1.010)	0.147	1.006 (1.001–1.011)	0.025
Cardiovascular disease^ab^	1.701 (1.211–2.388)	0.002		
Heart failure^a^	2.784 (1.507–5.142)	0.001		

HR, hazard ratio; CI, confidence interval; BMI, body mass index; SBP, systolic blood pressure; ACR, albumin to creatinine ratio; eGFR, estimated glomerular filtration rate; HbA1c, glycosylated hemoglobin; HDL-cholesterol, high-density lipoprotein cholesterol; LDL-cholesterol, low-density lipoprotein cholesterol.

^a^Defined as a comorbidity that a diagnostic code existed prior to study enrollment.

^b^Myocardial infarction, cerebrovascular accident, transient ischemic attack, and peripheral vascular disease were combined into a cardiovascular disease based on whether any of the individual comorbidities were present.

^c^Age, sex, BMI, SBP, duration of diabetes, stage of albuminuria, eGFR, HbA1c, HDL cholesterol, LDL cholesterol, and history of comorbidities (cardiovascular disease and heart failure) were included in the multivariate analysis.

Table 3 shows estimated hazards of all-cause mortality in elderly patients with diabetes. In univariate analyses, albuminuria was a strong risk factor for all-cause mortality. After adjusting for other covariates, both microalbuminuria and macroalbuminuria had 1.742 (1.411–2.153, *P* < 0.001) and 1.810 (1.344–2.441, *P* < 0.001) times the risk of all-cause mortality compared with normoalbuminuria. Dose–response relationships were not observed between albuminuria stages and hazard ratios of all-cause mortality. Furthermore, the increased risk of albuminuria for all-cause mortality was consistent in all the three age groups (Fig. 3b). No significant interactions were observed between albuminuria stages and age groups.

**Table 3:** Risk factors of all-cause mortality in elderly (≥ 65 years) patients with diabetes.

Variables	Univariate analysis		Multivariate analysis^c^	
	HR (95% CI)	*P* value	HR (95% CI)	*P* value
Age, year	1.122 (1.107–1.138)	< 0.001	1.101 (1.085–1.118)	< 0.001
Female (vs. male)	0.699 (0.591–0.826)	< 0.001	0.670 (0.565–0.795)	< 0.001
BMI, kg/m^2^	0.962 (0.934–0.992)	0.013	0.963 (0.938–0.990)	0.007
SBP, mmHg	1.004 (0.998–1.011)	0.212		
Duration of diabetes (per 1 year)	1.020 (1.011–1.029)	< 0.001		
ACR < 30 mg/gCr	1		1	
ACR 30 to 300 mg/gCr	2.321 (1.941–2.775)	< 0.001	1.742 (1.411–2.153)	< 0.001
ACR > 300 mg/gCr	2.632 (2.072–3.344)	< 0.001	1.810 (1.344–2.441)	< 0.001
eGFR, ml/min/1.73 m^2^	0.972 (0.967–0.977)	< 0.001	0.988 (0.982–0.993)	< 0.001
HbA1c, %	1.158 (1.076–1.246)	< 0.001	1.152 (1.074–1.236)	< 0.001
HDL cholesterol, mg/dl	0.993 (0.986–1.000)	0.045		
LDL cholesterol, mg/dl	0.998 (0.995–1.001)	0.163		
Cardiovascular disease^ab^	2.079 (1.753–2.465)	< 0.001	1.462 (1.226–1.743)	< 0.001
Heart failure^a^	2.609 (1.870–3.641)	< 0.001	2.115 (1.501–2.980)	< 0.001

HR, hazard ratio; CI, confidence interval; BMI, body mass index; SBP, systolic blood pressure; ACR, albumin to creatinine ratio; eGFR, estimated glomerular filtration rate; HbA1c, glycosylated hemoglobin; HDL-cholesterol, high-density lipoprotein cholesterol; LDL-cholesterol, low-density lipoprotein cholesterol.

^a^Defined as a comorbidity that a diagnostic code existed prior to study enrollment.

^b^Myocardial infarction, cerebrovascular accident, transient ischemic attack, and peripheral vascular disease were combined into a cardiovascular disease (CVD) based on whether any of the individual comorbidities were present.

^c^Age, sex, BMI, SBP, duration of diabetes, stage of albuminuria, eGFR, HbA1c, HDL cholesterol, LDL cholesterol, and history of comorbidities (cardiovascular disease and heart failure) were included in the multivariate analysis.

Sensitivity analysis was performed to confirm the robustness of the results. We repeated the Cox regression analyses, using eGFR as a categorical variable or using the CCI score instead of specific comorbidities such as CVD and HF. Albuminuria remained a strong risk factor for incident ESKD and all-cause mortality after adjusting for other covariates. To evaluate the impact of albuminuria on long term outcomes among very elderly patients aged ≥ 80, we conducted Cox regression analyses for composite outcomes of ESKD and all-cause mortality. The adjusted hazard ratio of microalbuminuria compared with normoalbuminuria was 2.100 (1.357–1.356, *P* = 0.001) (Supplement Table 1).

## Discussion

In this longitudinal study, we investigated the clinical predictors of ESKD and all-cause mortality in elderly patients by focusing on albuminuria status. Our results are as follows: microalbuminuria was an independent risk factor for both ESKD and mortality even among elderly patients with diabetes after adjusting for other related covariates. In the subgroup analyses according to age, the associations between albuminuria and poor outcomes were not attenuated across different age groups and were preserved in elderly patients aged ≥ 75. These findings suggested that microalbuminuria should not be overlooked in elderly patients with diabetes.

Albuminuria is a robust risk factor of poor kidney outcome in patients with diabetes^14,15^. In a Japanese cohort study including 4328 patients with diabetes, microalbuminuria or macroalbuminuria was a strong predictor of ESKD at any stages of GFR^16^. In a large cohort of patients with diabetic in Kidney Early Evaluation Program (KEEP) study, albuminuria was also independently associated with progression to ESKD, and the hazard increased from 6.4 times in patients with microalbuminuria to 15.1 times in patients with macroalbuminuria^17^. However, the existing literature provides limited and conflicting information on the prognostic value of microalbuminuria in older patients. Several landmark randomized controlled trials (RCTs) have focused on the protective effects of RAAS blockade against DKD progression, and differences in kidney outcomes according to the extent of albuminuria have been mentioned^14,18–20^. However, most of these studies have been limited because elderly patients older than 70 years were excluded or comprised a small proportion of study subjects.

In our study targeting elderly patients, during a median follow-up duration of 97 months, microalbuminuria remained a potent risk factor for ESKD; this finding was also consistent in a subgroup analysis divided by age. These results support that, even in elderly patients, microalbuminuria and macroalbuminuria play independent roles in the decline of kidney function. Several studies have investigated the effects of albuminuria on kidney outcomes in elderly patients with diabetes. In a small study of elderly patients older than 75 years, including 94 patients with diabetic CKD and 146 patients with non-diabetic CKD, there was no difference in the ratio of the subsequent decline in kidney function when proteinuria was less than 1 g/L^21^. A study of relatively elderly patients with DKD cohorts with a mean age of 69 years demonstrated that proteinuria of 0.15–0.49 g/day (considered comparable to microalbuminuria) is not a risk factor for ESKD^22^. However, the present study with a longer follow-up duration and a greater number of study subjects showed that microalbuminuria was associated with a 3.5-fold higher risk of progression to ESKD, and macroalbuminuria was associated with a tenfold higher risk of progression to ESKD.

Many previous studies have demonstrated that albuminuria is a predictor of cardiovascular risk and mortality in elderly patients with diabetes^12,13,23,24^. Tancredi et al.^12^ have reported that microalbuminuria and macroalbuminuria are associated with all-cause mortality and cardiovascular mortality and that the magnitude of the risk tended to decrease from the younger group to the very elderly group. Another diabetic cohort study reported that both microalbuminuria and macroalbuminuria are independently associated with increased risk of death at all GFR levels in patients aged ≥ 75 years, and similar results were obtained except for severely reduced GFR in patients aged 65–74 years^13^. Consistently, the present study showed that microalbuminuria and macroalbuminuria were associated with a 1.7 to 1.8-fold increase in the risk of all-cause mortality in elderly patients with diabetes. A dose–response relationship was not observed between albuminuria stages and all-cause mortality.

A number of studies have reported that anti-proteinuric therapies through RAAS blockade attenuate the risk of cardiovascular death and all-cause death in the general population with diabetes^25,26^. However, whether this applies to elderly patients remains unknown. The use of RAAS blockade occasionally triggers episodes of orthostatic hypotension, hyperkalemia, or acute kidney injury^27^, which are common in elderly patients due to physiological and anatomical alterations and complex comorbidities^28,29^. When we investigated medications in each subject, more than 60% of the present study population used ACEIs or ARBs; this was a higher percentage than that reported in another study of elderly patients^12^. In addition, no difference was observed in the frequency of ACEIs or ARBs prescriptions among the three age groups within the same albuminuria stages. Considering that the proportion of patients receiving statins was also high, our study population probably received active treatment, regardless of age. When comparing albuminuria grades, ACEIs or ARBs were similarly prescribed for patients with microalbuminuria, but were prescribed with a higher frequency for those with macroalbuminuria than for those with normoalbuminuria (65.0%, 69.0% vs. 64.7%). These findings suggest that the prognostic significance of microalbuminuria at least in elderly patients, may have been overlooked.

This study has several limitations. First, the retrospective observational study, which was performed via an electronic medical record (EMR) review, was not able to remove all potential confounders. However, this study design and the supplementation of almost all data about dialysis and death from the Korean Society of Nephrology (KSN) and government departments allowed us to obtain data with a large sample size and long-term follow-up. Eventually, we could evaluate the impact of albuminuria stage on ESKD and all-cause mortality among elderly patients with diabetes. Second, the study was conducted at a single center in a large urban teaching hospital. Among the study subjects, 70.4% had preserved kidney function, and 65.2% had normoalbuminuria. These proportions were higher than those observed in other longitudinal observational studies of diabetes^30,31^. In other words, the study participants probably included relatively healthier elderly individuals with diabetes. Thus, the results might be limited in application to the general elderly patients with diabetes. However, we believe that our results are more relevant to stable patients under optimal treatments, as we enrolled most of the elderly patients attending outpatient clinics regularly for 1 year. Third, sodium-glucose cotransporter-2 inhibitors and glucagon like peptide-1 receptor agonist, which have proven positive effects on cardiovascular outcomes in recent years, were not included as covariates in present study. At the first half of follow-up, both medications were prescribed in less than 1.0%. So, we concluded that it was difficult to evaluate the effect of the medications on patients’ prognosis through this study. Furthermore, serum creatinine level and urine albumin-to-creatinine ratio (ACR) were based on single measurements in this study, accompanied by misclassification bias. However, misclassification tends to attenuate this association, and the strong association between albuminuria and increased risk of ESKD and mortality is worthy of recognition in this study.

In conclusion, albuminuria is a strong dose-dependent risk factor for incident ESKD in elderly patients with diabetes. In all age groups, the impact of albuminuria was consistent after adjusting for eGFR and other covariates. In addition, microalbuminuria was associated with all-cause mortality, and the magnitude of the risk did not attenuate with aging. These findings suggest that even microalbuminuria is an important clinical predictor of patient and kidney outcomes in elderly patients with diabetes. Further studies are needed to determine whether treatment to reduce albuminuria is cost-effective in this population.

## Methods

### Study design and population

In this retrospective cohort study, we enrolled elderly patients (≥ 65 years) who were regularly followed up for type 2 diabetes in 2009 at the Samsung Medical Center, Seoul, Korea. Type 2 diabetes was defined as an HbA1c of greater than or equal to 6.5%, or the use of diabetes medications, including insulin. Regular follow-up was defined as visits to the endocrinology or nephrology outpatient clinic more than three times during 1 year. We excluded patients whose urine ACR or eGFR data were unavailable for the whole year of 2009, with a history of hospitalization within a year prior to study enrollment, and with an eGFR value lower than 15 ml/min/1.73 m^2^ with or without replacement therapy. Finally, 3065 elderly patients with diabetes were included in the study. Baseline was defined as the time of the first urine ACR measurement in 2009. Patients were divided into three groups: 65–69 years, 70–74 years, and ≥ 75 years.

This study was approved by the Institutional Review Board (IRB) of Samsung Medical Center (No. 2017-02-016). The IRB allowed that the requirement for informed consent from the participants was waived because we used only de-identified data. All methods were performed in accordance with the relevant guidelines and regulations.

### Variables and definitions

Baseline demographic and laboratory data including age, sex, body mass index (BMI), systolic blood pressure (SBP), duration of diabetes, urine ACR, eGFR, HbA1c level, high-density lipoprotein-cholesterol (HDL-C) level, low-density lipoprotein-cholesterol (LDL-C) level, CCI, comorbidities, and treatments such as ARBs or ACEIs, statins, insulin, metformin, dipeptidyl peptidase-4 inhibitors, sulfonylureas, and α-glucosidase inhibitors were retrospectively retrieved from the EMR database. Treatment was defined as being prescribed for more than a year. The International Classification of Diseases 10th revision (ICD-10) codes were used to define comorbidities and to score the CCI^32,33^. Myocardial infarction, cerebrovascular accident, transient ischemic attack, and peripheral vascular disease were combined with CVD based on the presence of any individual comorbidity. Codes for HF and end-organ damage (diabetic retinopathy, diabetic neuropathy, and albuminuria) that existed prior to study enrollment were also collected. Baseline eGFR was calculated using the Chronic Kidney Disease Epidemiology Collaboration (CKD-EPI) equation using the earliest outpatient serum creatinine measurement obtained in 2009^34^. Albuminuria stages were categorized as follows: (1) normoalbuminuria (A1) if the ACR was < 30 mg/gCr; (2) microalbuminuria (A2) if the ACR was 30 to 300 mg/gCr; or (3) macroalbuminuria (A3) if the ACR was > 300 mg/gCr. A2 and A3 were collectively regarded as having albuminuria (ACR ≥ 30 mg/gCr).

### Outcomes

Patients were followed from baseline to either the date of death or July, 26, 2017. Mortality data were obtained from the Ministry of the Interior and Safety, which included virtually all information about the deaths of Koreans. Additionally, we identified incident ESKD through the EMRs of the patients who started replacement therapy at our hospital. For patients who were lost to follow-up at our hospital before the initiation of replacement therapy, information about the application of replacement therapy was obtained from the KSN registry data on February 26, 2017.

### Statistical methods

Continuous variables are presented as mean ± standard deviation (SD) or median and interquartile range (IQR) based on their distribution, and categorical variables are presented as percentages. Variables were compared using ANOVA or the Kruskal–Wallis test for their characteristics.

The cumulative incidence of ESKD and all-cause mortality rate were estimated using Kaplan–Meier methods according to albuminuria stages and were compared using Cox proportional hazard regression considering multiple comparisons with Bonferroni correction because the number of patients stratified by albuminuria stage was markedly unbalanced. We calculated the cumulative incidence of ESKD considering the competing risk because ESKD and death are competitive events.

To estimate the risks of ESKD and all-cause mortality for each albuminuria stage, Cox proportional hazard regression models were used. Age, sex, BMI, SBP, duration of diabetes, stage of albuminuria, eGFR, HbA1c, HDL cholesterol, LDL cholesterol, and history of comorbidities (cardiovascular disease and heart failure) were included in the multivariate model for both outcomes. Interactions between albuminuria stages and age groups were evaluated in models adjusted for covariates for incident ESKD and all-cause mortality.

Statistical significance was set at *P* < 0.05. Data were analyzed using SAS version 9.4 (SAS Institute, Cary, NC) and R 3.6.1 (Vienna, Austria; http://www.R-project.org/).

## Supplementary Information

Supplementary Table 1.

## Data Availability

The datasets generated and/or analysed during the current study are not publicly available due to the lack of approval from our ethics committee in this regard but are available from the corresponding author on reasonable request.
