# Supplementary material for: Prognostic significance of albuminuria in elderly of various ages with diabetes
Source: Sci Rep. 2023 May 1;13:7079. doi: 10.1038/s41598-023-32305-0 (PMC10151322; doi:10.1038/s41598-023-32305-0)
Supplement: Supplementary file 1 — Supplementary Table 1. [file 41598_2023_32305_MOESM1_ESM.docx]

**Supplement Table 1. Risk factors of composite outcome (end stage kidney disease or all-cause mortality) in very elderly (****≥80 years) patients with diabetes**

|  | **Univariate analysis** | | **Multivariate analysis**^c^ | |
| --- | --- | --- | --- | --- |
| **Variables** | **HR (95% CI)** | ***P* value** | **HR (95% CI)** | ***P* value** |
| Age, year | 1.135 (1.065-1.209) | <0.001 | 1.115 (1.045-1.189) | 0.001 |
| Female (vs. male) | 0.650 (0.444-0.950) | 0.026 | 0.705 (0.475-1.045) | 0.082 |
| BMI, kg/m^2^ | 0.963 (0.913-1.016) | 0.163 |  |  |
| SBP, mmHg | 0.995 (0.979-1.010) | 0.485 | 0.989 (0.974-1.005) | 0.167 |
| Duration of diabetes (per 1 year) | 1.002 (0.985-1.020) | 0.779 |  |  |
| ACR <30 mg/gCr | 1 |  | 1 |  |
| ACR 30 to 300 mg/gCr | 2.292 (1.521-3.455) | < 0.001 | 2.100 (1.357-1.356) | 0.001 |
| ACR >300 mg/gCr | 1.868 (0.951-3.668) | 0.069 | 1.610 (0.784-3.307) | 0.195 |
| eGFR, mL/min/1.73m^2^ | 0.975 (0.963-0.988) | < 0.001 | 0.979 (0.966-0.992) | 0.002 |
| HbA1c, % | 1.127 (0.956-1.329) | 0.154 | 1.144 (0.965-1.356) | 0.121 |
| HDL cholesterol, mg/dl | 0.985 (0.969-1.000) | 0.053 |  |  |
| LDL cholesterol, mg/dl | 1.003 (0.997-1.009) | 0.291 |  |  |
| Cardiovascular disease^ab^ | 1.198 (0.814-1.763) | 0.359 |  |  |
| Heart failure^a^ | 2.000 (0.971-4.122) | 0.060 |  |  |
| ^a^Defined as a comorbidity that a diagnostic code existed prior to study enrollment.  ^b^Myocardial infarction, cerebrovascular accident, transient ischemic attack, and peripheral vascular disease were combined into a cardiovascular disease (CVD) based on whether any of the individual comorbidities were present.  ^c^ Age, sex, BMI, SBP, duration of diabetes, stage of albuminuria, eGFR, HbA1c, HDL cholesterol, LDL cholesterol, and history of comorbidities (cardiovascular disease and heart failure) were included in the multivariate analysis.  Abbreviations: HR, hazard ratio; CI, confidence interval; BMI, body mass index; SBP, systolic blood pressure; ACR, albumin to creatinine ratio; eGFR, estimated glomerular filtration rate; HbA1c, glycosylated hemoglobin; HDL-cholesterol, high-density lipoprotein cholesterol; LDL-cholesterol, low-density lipoprotein cholesterol. | | | | |
